# Epigenetics and chemical exposures

**DOI:** 10.1042/EBC20253053

**Published:** 2025-12-24

**Authors:** Adriana Córdova-Casanova, Susan E. Ozanne

**Affiliations:** 1 University of Cambridge, Institute of Metabolic Science and Medical Research Council Metabolic Diseases Unit, Addenbrooke’s Hospital, Cambridge, U.K

**Keywords:** DNA, epigenetics, histones

## Abstract

Seminal epidemiological studies led by Barker, Hales and collaborators identifying links between low birth weight and subsequent risk of traditionally adult-onset diseases such as cardiovascular disease and type 2 diabetes led to the concept of what is now termed the Developmental Origins of Health and Disease (DOHaD). This suggests that suboptimal exposures in early life (including the *in utero* period) permanently influence the structure, physiology, biochemistry and molecular biology of our organ systems and therefore our long-term health. Although the initial epidemiological observations almost 40 years ago focussed on the consequences of nutrient restriction during early life on long-term cardiometabolic health, the DOHaD field has now established a much broader spectrum of suboptimal exposures (including chemicals and environmental toxins) that affect a wide range of health outcomes (including certain forms of cancer and mental health). Epigenetic processes are thought to play an important mechanistic role in mediating the effects of a suboptimal *in utero* environment on long-term health. This includes changes in DNA methylation and histone modifications that regulate gene expression without affecting the DNA sequence. This special issue focuses on current knowledge on the impact of chemical exposures, such as heavy metals and endocrine disruptors, on the epigenome and long-term health outcomes, including mental health disorders, cardiovascular and neurodegenerative diseases.

## Early life exposures, epigenetics and long-term health

Studies of individuals who were exposed to periods of famine whilst *in utero*, such as survivors of the Dutch Hunger Winter and the Chinese Great Leap Forward famine, provided some of the earliest evidence that suboptimal exposures during fetal life affected long-term health [1–3]. These studies demonstrated that it was not only cardiometabolic health that was influenced by *in utero* exposures, but also outcomes such as mental health, including conditions such as schizophrenia. The molecular mechanisms underlying these relationships remain to be fully understood. However, there has been much attention directed to the role of epigenetics. This term, meaning ‘on top of genetics’, was first used by Waddington in 1940 as a concept [4] to explain how non-sequence modifications of the genome modulate the expression of genes. Epigenetics is now used as a term that encompasses changes in DNA methylation, histone modifications and the levels of non-coding RNAs. Early life environmental factors have been associated with persistent changes in the epigenome in humans [5]. These studies have also highlighted that it is not only nutritional factors in early life that permanently influence the epigenome, but that other factors such as endocrine disruptors and chemicals can also have a long-term impact [6,7]. They have also demonstrated that different factors in the *in utero* and perinatal periods influence the epigenome but that they can also emerge later in life in response to environmental conditions. Proof of concept for this can be observed when comparing the DNA methylome of monozygotic twins, where it has been demonstrated that the differences in DNA methylation become greater with age between the two twins [8].

## Environmental exposures and human disease

In 2016, the World Health Organization highlighted that exposure to environmental chemicals, including air pollution, heavy metals and endocrine disruptors impairs human health and that these represent a risk for chronic conditions [9]. They estimated that 24% of total deaths and 28% of deaths in children under five in the world could be attributable to modifiable environmental factors [9]. As human environments are constantly changing, due to economic, social or climate determinants, there is an important need to be responsive to assessing the impact of emerging new environmental exposures and ensuring that such exposures are addressed in different settings. In this issue, Dewey and Hussain [10] summarise recent studies, from a range of settings, demonstrating effects of exposure to heavy metals such as arsenic, cadmium, lead and mercury during prenatal and early life on children’s poor neurodevelopment and mental health. On a related topic but focussing on longer-term outcomes, Antinori and colleagues [11] review recent findings supporting a causal link between arsenic exposure and Alzheimer’s disease and the potential contribution of epigenetic mechanisms through effects of oxidative stress on neurotoxicity. Lin and Zhou [12] review the evidence for the impact of endocrine disruptors such as DDT, PCDDs, PCBs and BPA on epigenetic modifications that could contribute to the development of atherosclerosis and metabolic diseases. Jirtle [13] highlights two epigenetically labile groups of genes (those that undergo genomic imprinting and those with metastable epialleles) that could link environmental exposures during early life with long-term risk of both chronic diseases and behavioural disorders in adulthood. These four mini reviews therefore provide a comprehensive overview of the effect of the most common chemical exposures and their interactions with the epigenome and negative impacts on human health.

## Epigenetic tools to predict and prevent disease

Ageing is a physiological process strongly associated with increased risk of chronic diseases. Accelerated molecular, cellular and physiological ageing has been identified as one potential mechanism to link suboptimal early life exposures and long-term disease risk. Therefore, determining the biological over the chronological age could be a tool to predict disease risk or even monitor public health in the context of environmental chemical exposures. In this special issue, England-Mason [14] discusses the potential use of epigenetic clocks as an ageing biomarker to estimate biological age in perinatal and paediatric samples as a consequence of exposure to environmental chemicals. With cumulative evidence demonstrating that environmental exposures can affect the epigenome and that these changes exhibit strong correlations with various human diseases, a key question arises regarding the possibility of reversing epigenetic modifications as a potential therapeutic approach. In this issue, Allen and Guerrero-Bosagna [15] review the current evidence for the reversibility of changes in epigenetic marks induced by chemical exposures and the potential for such an approach in the treatment of mental health disorders.

## Summary and future perspectives

The mini reviews compiled in this issue summarise some of the recent scientific progress that has been made in the study of chemical exposures and epigenetics and their potential role in contributing to shaping human biology, behaviour and health outcomes. Although much progress has been made in moving from association studies to those showing causal relationships, it remains challenging, at a population level, to dissect out the causative effects of specific exposures on defined epigenetic loci to mediate effects on health outcomes. There has been important progress in using epigenetic modifications as biomarkers of both suboptimal exposure and disease risk. This paves the way for the use of potential new ‘Epidrugs’ both in the treatment of human disease and in preventative medicine. The reversibility of epigenetics marks as an approach for new therapeutic targets and will therefore undoubtedly be an area that we hear much more about in the coming years.

## Abbreviations

BPA: bisphenol A
DDT: dichloro-diphenyl-trichloroethane
DOHaD: Developmental Origins of Health and Disease
PCBs: polychlorinated biphenyls
PCDDs: polychlorinated dibenzodioxins
